# Organizing the health interview survey at the local level: design of a pilot study

**DOI:** 10.1186/s13690-022-00909-z

**Published:** 2022-06-10

**Authors:** Lize Hermans, Elise Braekman, Sabine Drieskens, Stefaan Demarest

**Affiliations:** Sciensano, Scientific Direction Public Health and Epidemiology, Juliette Wytsmanstraat 14, B-1050 Brussels, Belgium

**Keywords:** Health interview survey, Belgium, Survey methodology, Municipality

## Abstract

**Background:**

The local Health Interview Study (LHIS) was developed to gain health information at the level of the municipality in Flanders, the northern part of Belgium. It enables municipalities to make evidence-based decisions in their public health policy. To test the feasibility of implementing the LHIS, a pilot study was conducted in Melle, a small Flemish municipality with 11.736 inhabitants.

**Methods:**

The target sample size was 1000 (≥ 15 years). A systematic sampling technique was applied with substitutes for non-respondents who were matched in terms of statistical sector, age and sex. Selected persons were contacted by post to complete the questionnaire and in case of non-response, a reminder was sent. Questionnaires were collected using a concurrent mixed-mode design: a paper and pencil, and web option. All questions were selected from the Belgian Health Interview Survey relating to health status and determinants of health.

**Results:**

One thousand twenty-two questionnaires were obtained after inviting 3137 individuals (response rate = 32.6%). Older adults were more likely to participate than younger adults, and women more than men. The final sample resembled the initial sample in terms of sex and statistical sector, but not in terms of age. Younger adults were underrepresented whereas older adults were overrepresented. Lastly, older adults were more likely to fill in the questionnaire on paper than younger adults, and women more than men.

**Conclusion:**

The LHIS can be successfully implemented in Flemish municipalities. The method, however, does not guarantee that the composition of the final sample reflects the initial sample. Therefore, weights should be added in the analyses to correct for potential deviations in sample composition. Furthermore, implementing a sequential mixed-mode design with a web option preceding a paper and pencil option in future studies could reduce costs and improve data quality.

**Supplementary Information:**

The online version contains supplementary material available at 10.1186/s13690-022-00909-z.

## Background

Gaining reliable information on public health is crucial for a tailored policy design. Health data on the national and regional level in Belgium is routinely being collected, however, a standardized method to measure health indicators at municipality level is lacking. To address this data gap, the Belgian Scientific Institute for Public Health (since 2018 Sciensano) was commissioned in 2017 by the Flemish Minister responsible for Welfare and Public Health to develop the Local Health Interview Survey (LHIS).

The purpose of the LHIS is to provide Flemish municipalities with information on various health-related topics with the emphasis on health and well-being, health behavior and lifestyle and health and society. This would enable them to make evidence-based decisions and set priorities in their local public health policy. The majority of the Flemish municipalities are enrolled in the ‘Healthy Municipality’ project [1] – a project based on the principle of subsidiarity [2] which is a “principled tendency toward solving problems at the local level and empowering individuals, families and voluntary associations to act more efficaciously in their own lives” (p116) [3]. Within this project a networking organization of the Flemish government called the Logos support the municipalities in shaping their health policy. They advise the municipalities on actions that can be taken and provide tools and materials for this purpose. For instance, they provide materials that municipalities can use to educate the elderly population about the risks of falling and ways to reduce the risk of falling. For many health indicators there is no information available on the local level and decision makers have to rely on regional data to set priorities. These data, however, are insufficient to guide local policy makers since not all municipalities are the same (e.g. differences in age composition, income, educational level, size, degree of urbanization etc.). This illustrates the need for obtaining health data locally. Population health on a regional level would also benefit from such a local-level approach.

The methods and content of the Belgian Health Interview Survey (BHIS) were taken as a starting point to develop a standardized tool for obtaining health data locally. The BHIS is the leading health survey in Belgium since 1997 [4] and is being repeated every 4 to 5 years with the latest survey in 2018 and a future survey planned in 2023. The BHIS has a cross-sectional design and its content has been increasingly aligned to the European Health Interview Survey (EHIS) [5] over the years. Data is collected through face-to-face interviews supplemented with a self-administered questionnaire for the more sensitive topics (e.g. mental health and use of illicit drugs). To reach a predefined net sample in terms of size and composition, sample substitution is applied [6]. Sample substitution is the process of replacing non-respondents by individuals with a similar profile in terms of predefined variables (e.g. sex, age) during the field work.

An important prerequisite of the LHIS is that it needs to be cost-effective and thus it would not be feasible to adopt the face-to-face approach of the BHIS. A web questionnaire can be a cost-effective alternative, however, it may potentially result in low response rates in a general population study [7, 8]. In light of this problem, Braekman et al. (2019) recommend using a concurrent mixed mode design including a web and paper and pencil (P&P) questionnaire [7]. This design might introduce measurement differences between the two modes but research has shown that the impact on health estimates is rather limited [9, 10]. Opting for a self-administered questionnaire also implies that the number of questions should be limited since break-off is much easier [11, 12]. The topics should be chosen based on their added value in a local setting, focusing on the health status of an individual and determinants of health (such as an individual’s background, well-being, lifestyle, and physical and social environment). By only selecting topics from the BHIS, the BHIS results for Flanders can be used as a benchmark.

Similar studies in terms of design (mixed-mode) and content (questions) can be found in the framework of the EHIS. Notwithstanding the recommendation for face-to-face interviewing [13, 14], member states show an increasing interest in mixed-mode designs with a web questionnaire [7, 15]. In the second wave of the EHIS, which took place between 2013 and 2015, 10 member states used a web questionnaires and four of them combined it with a P&P questionnaire (for an overview, see [7]). The response rates for these studies ranged between 26 and 48%.

To evaluate the feasibility of implementing the LHIS, a pilot study was performed in Melle, a small Flemish municipality. In 2019, it counted 11,736 inhabitants while the average Flemish municipality counted 21,964 inhabitants. A municipality with a small capacity in terms of resources (employees, budget, …) was chosen since one would assume that if they succeed in organizing the LHIS, a municipality with a larger capacity would be successful as well. The first objective of the present article is to describe the design and methodology of the LHIS. The second objective is to evaluate the response rate and sample composition in the pilot municipality.

## Methods

### Target population

The target population of the LHIS included all inhabitants of Melle who were at least 15 years of age, including those who turned 15 in the year of sampling. The sampling frame consisted of all persons enlisted in the Population Register of the municipality. As such, the study population of the LHIS included all inhabitants of the municipality regardless of their place of birth, nationality or any other characteristic. It did not include people living in Melle who were not listed in the Population Register (homeless, undocumented immigrants, etc.) and people whose date of birth was not officially known. Consequently, the study population did not perfectly cover the target population, which is also the case in the BHIS.

### Sampling scheme

The sampling frame was hierarchically ordered by the statistical sector (i.e. a small district in the municipality), age group and sex. The study population was divided in seven age groups, which corresponded to those in the BHIS: 15–24, 25–34, 35–44, 45–54, 55–64, 65–74 and ≥ 75 years.

Taking into consideration budget and logistical constraints, the sample size was set on 1000. Due to the uncertainty of the response rate in this survey, field substitution was chosen as a method to reach the predefined sample size. For every selected individual, three individuals with matching characteristics in terms of statistical sector, age and sex were selected as substitutes. These four individuals represent a cluster. In case of non-response, the next individual in the cluster was selected as substitute. To increase the speed of data collection, the initial sample was inflated and 1500 individuals instead of 1000 individuals were selected. If only 1000 individuals were to be invited, many waves would be needed to reach the net sample of 1000 individuals. As a result, there were 1500 clusters of four individuals with a similar sociodemographic profile. Every person in the sample was given a unique personal 6-digit identification number to monitor the response without using personal information.

### Questionnaire

The LHIS questionnaire was based on questions used in the BHIS 2018. Four different needs were taken into account for the specific selection of questions regarding the LHIS. First, questions were selected based on the interests of the involved parties and the relevance for the municipality. Second, the questionnaire was designed such that one can complete it without the intervention of an interviewer. As such, attention was given to the vocabulary and possible obstacles while filling in the questionnaire. Third, due to the limited sample there was a preference for questions addressing the whole population. Sub questions (i.e. addressed to a subset of the sample such as for example persons from a specific age group) were as much as possible avoided. The combination of sub questions and a limited sample may result in a limited power to detect differences between categories (e.g. educational levels). Finally, the time to complete the questionnaire was limited to 15 min to achieve a higher response rate.

Twenty-two topics were included: personal information (date of birth, sex and nationality), education, employment, subjective health, dental health, stress and well-being, vitality, quality of life, contact with general practitioner, frailty, alcohol consumption, tobacco smoking, e-cigarettes use, use of illicit drugs, physical activity, nutritional status, nutritional habits, health and environment, exposure to tobacco smoke, accidents and injuries, social health and informal care.

The data were collected in a concurrent mixed-mode design. The questionnaire could be completed on paper (P&P questionnaire, Additional file 1) or online (web questionnaire). Both questionnaires were only available in Dutch. They were identical, except for the instructions on how to complete it. The web questionnaire was developed in Blaise® 4.8.

### Fieldwork

Data were collected in different waves with a maximum number of four. Data collection started on April 13, 2018. At the start of the first wave, the municipality sent invitations by post to the first individuals in the 1500 clusters. The invitation contained a letter of the mayor, a personalized letter [16] of Sciensano, the paper questionnaire and a pre-addressed franked envelope to return the questionnaire. The letter of the mayor was included to encourage the candidates to participate and increase the response rate. The letter of Sciensano contained a web link to the online questionnaire and the unique 6-digit identification number, which participants needed to access the questions online. In case participants opted for the paper questionnaire, they had to write down the identification number such that the response could be monitored.

Fourteen days after sending the invitation, the municipality sent a reminder to all individuals who were invited but did not participate. The reminder contained only a letter of Sciensano.

After another 14 days, the response was evaluated again and a new data collection wave was started. In case of non-response, the next individual in the cluster (i.e. a substitute) was activated, while the initial selected person was deactivated and thus could no longer participate. This process was repeated until 1000 questionnaires were collected.

### Statistical analyses

A logistic regression model with age and sex as independent variables was used to analyze the response rate and the mode of response (P&P or web). Odds Ratios (OR) and 95% confidence intervals (CI) were calculated. To compare the composition of the initial sample (invited individuals in wave 1) with the one of the final sample based on the socio-demographic characteristics that were used in the sampling scheme (i.e. sex, age group and statistical sector), a chi-square test was used. The data analysis for this paper was generated using SAS software, Version 9.4. Copyright © 2013 SAS Institute Inc. SAS and all other SAS Institute Inc. product or service names are registered trademarks or trademarks of SAS Institute Inc., Cary, NC, USA.

## Results

Three data collection waves were performed during which 3137 inhabitants of Melle were invited to participate (Table 1). 1022 inhabitants completed the questionnaire (global response rate = 32.6%). Table 1 presents an overview of the response rate per wave. The data showed a decrease in response rate in subsequent data collection waves.

**Table 1:** Number of invitations, reminders, completed questionnaires, and the response rate per wave

Wave	Invitations (n)	Reminders (n)	Completed questionnaires (n)	Response rate (%)
1	1500	1111	518	34.53
2	982	760	327	33.30
3	655	540	177	27.02
Total	3137	2411	1022	32.58

The answers on the questions assessing sex and age were compared to the information of the Population Register. A total of 18 questionnaires (1,8%) were filled out by a different person than the one selected from the Register. These respondents were excluded from further analyses.

Response rates differed across age and sex (Table 2). More specifically, older adults were more likely to respond than younger adults (OR = 1.022, 95% CI [1.018, 1.026], *p* < 0.001). Men were less likely to respond than women (OR = 0.838, 95% CI [0.718, 0.977], *p* = 0.0243).

**Table 2:** Sample size, response rates and mean age of all respondents, and number, percentages and mean age of respondents who opted for the P&P questionnaire

Characteristics	Respondents		Mode: P&P	
	**n**	**% / mean**	**n**	**% / mean**
**All**	1004	32.2%	692	68.9%
**Sex**				
**Men**	452	29.6%	283	62.6%
**Women**	552	34.7%	409	74.1%
**Age**	1004	52.8 years	692	56.4 years
**Age group (in years)**				
**15—24**	94	20.8%	54	57.5%
**25—34**	118	23.2%	58	49.2%
**35—44**	138	26.0%	80	58.0%
**45—54**	165	32.4%	109	66.1%
**55—64**	175	37.1%	120	68.6%
**65—74**	156	51.3%	123	78.9%
** ≥ 75**	158	46.1%	148	93.7%

Of the respondents, 68.9% opted for the P&P questionnaire. Table 2 gives a summary of the percentage of completed P&P questionnaires by sex and age and the mean age of P&P respondents. Age and sex had a significant effect on the mode of completion. Older adults were more likely to opt for the P&P questionnaire than younger adults (OR = 1.034, 95% CI [1.026, 1.042], *p* < 0.001). Men were less likely than women to opt for the P&P questionnaire (OR = 0.538, 95% CI [0.406, 0.714], *p* < 0.001).

Table 3 gives a summary of the composition of the initial and final sample. The final sample resembled the initial sample in terms of sex (*χ*^*2*^ (1) = 3.110, *p* = 0.0778) and statistical sector (*χ*^*2*^ (5) = 2.744, *p* = 0.739), but not in terms of age (*χ*^*2*^ (6) = 40.157, *p* < 0.001). Younger adults were underrepresented, whereas older adults were overrepresented.

**Table 3:** Composition of the initial and final sample

	**Initial sample** **(*n* = 1500)**		**Final sample** **(*n* = 1004)**		
	n	%	n	%	*p*^a^
**Sex**					.0778
**Men**	717	47.8	452	45.0	
**Women**	783	52.2	552	55.0	
**Age group (in years)**					< .001
**15—24**	194	12.9	94	9.4	
**25—34**	218	14.5	118	11.8	
**35—44**	243	16.2	138	13.8	
**45—54**	241	16.1	165	16.4	
**55—64**	236	15.7	175	17.4	
**65—74**	175	11.7	156	15.5	
** ≥ 75**	193	12.9	158	15.7	
**Statistical sector**					.739
**Centrum**	786	52.4	536	53.4	
**Dries**	5	0.3	4	0.4	
**Gontrode kern**^b^	1	0.1	0	0	
**Kloosterwijk**	13	0.9	7	0.7	
**Lindenhoek – L**	12	0.8	4	0.4	
**Melle – Gontrode**	127	8.5	85	8.5	
**Vogelhoek**	556	37.1	368	36.7	

^a^*p* values derived from chi-squared test

^**b**^This category was excluded from the analysis since there were no observations in this category in the final sample

The nationality and educational level of the final sample was evaluated by analyzing the answers to the questionnaire (Table 4). A total of 2.8% of the participants did not have the Belgian nationality, which is similar to the population of Melle in 2011 (2.7%) [17]. Half of the participants had a higher education degree or was enrolled in higher education at the time of the study. Of the participants of 20 years and older, 47.7% obtained a diploma of higher education, which is more than in the population of Melle in 2011 (36.5%) [17].

**Table 4:** Nationality and educational level of the final sample

	**n**	**%**
**Nationality**		
Belgian	975	97.1
Non-Belgian	28	2.8
Missing	1	0.1
**Educational level**		
No diploma/primary education	88	8.8
Lower secondary education	124	12.4
Upper secondary education or post-secondary non-tertiary education	259	25.8
Higher education	497	49.5
Missing	36	3.6

## Discussion

The LHIS is a cross-sectional postal survey with a mixed-mode web – P&P design that is developed to monitor health at the level of the Flemish municipality. In 2018, a LHIS pilot was tested in Melle, a small municipality with limited capacity in terms of resources.

Overall, one in three invited individuals completed the questionnaire. The response rate decreased in each subsequent data collection wave. This result was expected based on previous research [7, 8] and suggests that hard to reach individuals are replaced by similar individuals. Furthermore*,* our data showed that younger adults were less likely to respond than older adults, and men were less likely to respond than women which is in good agreement with previous studies [15, 18–21].

To ensure that the composition of the final sample reflected the initial sample in terms of age and sex, field substitution was applied. Nevertheless, we found that the final sample differed from the initial sample in terms of age, with a slight underrepresentation of younger adults and overrepresentation of older adults. This was likely a result of inflating the initial sample size and inviting more individuals than the net target. Therefore, it is still important to use weights in the analyses of the population data. The sampling frame (i.e. the Population Register) did not contain information about educational level or income and therefore it was not possible to compare respondents with non-respondents based on these socio-economic variables. Compared to the population of the pilot municipality in 2011, however, a larger share of participants completed higher education (i.e. a difference of 11%). This is in line with previous studies showing that people with a higher socio-economic background are more likely to participate [22–24]. In light of these findings, it is advisable to include education in the sampling scheme. This information could be obtained by linking data of different sources, in this setting the Population/National Register and the Administrative Census.

In the present study, a concurrent mixed-mode design was used: the invitation letter contained two different modes to complete the questionnaire, namely P&P and web. Almost 7 out of 10 participants filled in the paper questionnaire. It might have been easier to fill in the questionnaire on paper directly than to search for it online first. However, participants still had to take the effort of mailing the letter when filling it in on paper. In terms of data processing, the web questionnaire is preferred to the P&P since the answers of the completed questionnaires in the P&P mode require manual data-entry. This entails a specific budget and staff, and the risk of possible coding errors is higher compared to the web mode [25]. Nevertheless, it is necessary to present the P&P mode option because not all candidates have internet access, experience with the use of internet [26], or want to participate online e.g. because they do not trust the privacy connection. Furthermore, web only data collection may result in low response rates [7, 8] and underrepresentation of certain subgroups such as lower educated people [7] or elderly [27]. Our results also demonstrate a strong preference for the P&P mode in elderly, corroborating previous findings [28–30]. To try to increase the percentage of web completed questionnaires in future studies, an alternative design should be considered in which the P&P option is only offered in the reminder letter – i.e. a sequential mixed-mode design [31, 32].

Even if a municipality has the capacity in terms of resources to organize a local health survey, in some municipalities recruiting the target sample may be more challenging than in the pilot municipality. This might be particularly the case in municipalities with a larger population and with a higher proportion of persons with a migration background among the population for whom language might be a barrier. Consequently, it is advisable to make at least the web questionnaire available in more than one language as was the case now. In case of a lower response rate, a fourth wave of data collection can be conducted, in which the third replacements can be invited. Such a fourth wave was also foreseen in the present study but was not carried out since the target sample had been reached after the third wave.

The Population Register of the municipality was used as sampling frame in the pilot study. The municipality is responsible for maintaining and updating the Population Register which can be used for sampling after approval of the municipal council. According to the general data protection regulation law, however, it is not allowed to use the Population Register when multiple municipalities wish to organize the LHIS. In this case, the LHIS is considered to be of supra-local importance. Consequently, the National Register should be used instead of the Population Register as the sampling frame. The National Register contains information of all Belgians and its source of input is the Population Register of each municipality. To access the National Register, authorization should be obtained from the Sectoral Committee of the National Register.

The LHIS was successfully conducted in a pilot municipality with a response rate of one in three. In the final sample, the younger adults were underrepresented which should be taken into account in further analyses. In the future, the design as described in this article will be used as a basis to implement the LHIS in Flemish municipalities who wish to gain health information locally. When the LHIS will be rolled out, however, it will be necessary to change the source for the sampling frame and it might be worthwhile to provide the participants with more than one language option. To reduce the cost and improve the data quality, it should be considered to implement a sequential mixed-mode design with a web option preceding a P&P questionnaire.

## Conclusion

In Belgium, health information at the level of the municipality is lacking. To address this gap, a concurrent mixed-mode health survey (web/P&P) was designed based on the BHIS, i.e. the LHIS. In 2018, the LHIS was implemented in Melle, a small Flemish municipality with limited capacity. It can be concluded that it is feasible and worthwhile to organize a health survey at the level of the municipality in Belgium. Based on LHIS data, municipalities can evaluate the implementation of local health campaigns and interventions, and improve their effectiveness. Furthermore, they can compare their results with those of Flanders in the BHIS or other participating municipalities.

## Supplementary Information

**Additional file 1.**

## Data Availability

The dataset used and/or analyzed during the current study is available from the corresponding author following a request to the Health Interview Survey team at Sciensano and a signature of a Data Transfer Agreement.

## Abbreviations

BHIS: Belgian Health Interview Survey
CI: Confidence Interval
EHIS: European Health Interview Survey
LHIS: Local Health Interview Survey
OR: Odds Ratio
P&P: Paper and Pencil
