# Supplementary material for: Organizing the health interview survey at the local level: design of a pilot study
Source: Arch Public Health. 2022 Jun 10;80:155. doi: 10.1186/s13690-022-00909-z (PMC9185910; doi:10.1186/s13690-022-00909-z)
Supplement: Supplementary file 1 — Additional file 1. [file 13690_2022_909_MOESM1_ESM.pdf]

LOKALE GEZONDHEIDSENQUÊTE
MELLE 2018

Fijn dat u wil deelnemen aan deze studie. U hebt ervoor gekozen om de vragenlijst op papier in te vullen, u hoeft deze dus niet meer online in te vullen.

Wij garanderen u dat uw antwoorden volledig **anoniem en vertrouwelijk** verwerkt zullen worden. Gelieve zo correct en eerlijk mogelijk te antwoorden. Vul hieronder uw **persoonlijke toegangscode** in. Deze kan u terugvinden op de uitnodigingsbrief die u heeft ontvangen. Het is **noodzakelijk** dat u uw persoonlijke toegangscode invult. De codes worden enkel gebruikt om de respons anoniem op te volgen.

Toegangscode:

2	0	1	8	-	9	0	9	0	-						

Indien u bijkomende vragen heeft of technische problemen ervaart bij het invullen van de vragenlijst, kan u steeds contact opnemen via e-mail: eef.delodder@sciensano.be of telefoon: 02/642.52.42 (van maandag tot en met vrijdag, telkens van 9 tot 12 uur en van 14 tot 17 uur).

Hoe deze vragenlijst invullen?

- Voor de meeste vragen moet u een kruisje plaatsen in het vakje dat best past bij uw antwoord. Tenzij anders aangegeven, kruis telkens slechts één antwoord aan per vraag en ga door met de vraag die volgt.

2. Wat is uw geslacht?

1 ☒ Man

2 ☐ Vrouw

- Soms moet u een getal in het daarvoor bestemde vakje schrijven.

20. Hoe groot bent u, zonder schoenen aan?

1	7	9	cm

- In sommige gevallen leidt uw antwoord er toe dat u een reeks vragen overslaat. De pijl verwijst u naar een vraag verder in de vragenlijst.

21. Bent u momenteel zwanger?

1 ☐ Ja

2 ☒ Neen ⇨ *Ga naar Vraag 23*

Persoonsinformatie

1. Wat is uw geboortedatum? dd/mm/jjjj

		/			/				

2. Wat is uw geslacht?

1 ☐ Man
2 ☐ Vrouw

3. Wat is uw huidige nationaliteit?

Meerdere antwoorden mogelijk

1 ☐ Belgisch
2 ☐ Frans
3 ☐ Nederlands
4 ☐ Duits
5 ☐ Italiaans
6 ☐ Spaans
7 ☐ Portugees
8 ☐ Pools
9 ☐ Turks
10 ☐ Marokkaans
11 ☐ Andere nationaliteit 1: ______________________________
12 ☐ Andere nationaliteit 2: ______________________________

Studies

04. Bent u schoolgaand, d.w.z. volgt u (eventueel deeltijds) dagonderwijs?

- ☐ 1 Ja
- ☐ 2 Neen ⇨ *Ga naar Vraag 6*

05. Welk van de volgende studierichtingen volgt u op dit ogenblik?

- ☐ 1 Lager onderwijs
- ☐ 2 Lager secundair onderwijs of secundair onderwijs van de 1ste of 2de graad
- ☐ 3 Hoger secundair onderwijs of secundair onderwijs van de 3de graad
- ☐ 4 Post-secundair niet-hoger onderwijs (4° graad, 7de jaar, ondernemingsopleiding,...)
- ☐ 5 Hogescholenonderwijs van het korte type, graduaat (A1), professionele bachelor
- ☐ 6 Hogescholenonderwijs van het lange type, master aan een hogeschool
- ☐ 7 Academische bachelor (hogeschool of universiteit)
- ☐ 8 Universitair onderwijs, licentiaat, ingenieur of master
- ☐ 9 Doctoraat met proefschrift
- ☐ 10 Andere studierichting: ______________________________

06. Welk is het hoogste diploma of de hoogste graad die u behaald heeft?

- ☐ 1 Geen diploma
- ☐ 2 Lager onderwijs
- ☐ 3 Lager secundair onderwijs of secundair onderwijs van de 1ste of 2de graad
- ☐ 4 Hoger secundair onderwijs of secundair onderwijs van de 3de graad
- ☐ 5 Post-secundair niet-hoger onderwijs (4° graad, 7de jaar, ondernemingsopleiding,...)
- ☐ 6 Hogescholenonderwijs van het korte type, graduaat (A1), professionele bachelor
- ☐ 7 Hogescholenonderwijs van het lange type, master aan een hogeschool
- ☐ 8 Academische bachelor (hogeschool of universiteit)
- ☐ 9 Universitair onderwijs, licentiaat, ingenieur of master
- ☐ 10 Doctoraat met proefschrift
- ☐ 11 Ander diploma: ______________________________

Tewerkstelling

07. Hebt u op dit moment betaald werk, eventueel tijdelijk onderbroken?

- ☐ 1 Ja, betaald werk (eventueel tijdelijk onderbroken) ⇨ *Ga naar Vraag 9*
- ☐ 2 Neen, geen betaald werk

08. U verricht momenteel geen betaald werk. In welk van de volgende situaties bevindt u zich?

- ☐ 1 Werkloosheid
- ☐ 2 Ziekte of invaliditeit
- ☐ 3 Studies
- ☐ 4 Pensioen
- ☐ 5 Ik doe het huishouden, zonder uitkering
- ☐ 6 Ik ben helpster van een zelfstandige
- ☐ 7 Andere situatie: ____________________

Uw gezondheidsbeleving

09. Hoe is uw gezondheidstoestand in het algemeen?

- ☐ 1 Zeer goed
- ☐ 2 Goed
- ☐ 3 Gaat wel (redelijk)
- ☐ 4 Slecht
- ☐ 5 Zeer slecht

10. Hebt u een langdurige ziekte of aandoening (gezondheidsprobleem)?

- ☐ 1 Ja
- ☐ 2 Neen

11. Bent u, vanwege een gezondheidsprobleem, <u>sinds 6 maanden of langer</u> beperkt geweest in activiteiten die mensen gewoonlijk doen?

- ☐ 1 Ja, erg beperkt
- ☐ 2 Ja, beperkt
- ☐ 3 Neen, niet beperkt

Contacten met een huisarts

12. **Hebt u een vaste huisarts of een vaste huisartsengroepspraktijk? Het kan ook gaan om een wijkgezondheidscentrum.**

- ☐ 1 Ja
- ☐ 2 Neen

Mondgezondheid

13. **Hoe vaak poetst u gewoonlijk uw tanden?**

- ☐ 1 Meer dan twee keer per dag
- ☐ 2 Twee keer per dag
- ☐ 3 Eén keer per dag
- ☐ 4 Minder dan één keer per dag
- ☐ 5 Nooit

14. **Hoe zou u, in het algemeen, de gezondheidstoestand van uw tanden en kaken omschrijven?**

- ☐ 1 Zeer goed
- ☐ 2 Goed
- ☐ 3 Gaat wel (redelijk)
- ☐ 4 Slecht
- ☐ 5 Zeer slecht

Levenskwaliteit

Vink onder elke titel het ENE vakje aan dat het best uw gezondheid VANDAAG beschrijft

15. Mobiliteit

- ☐ 1 Ik heb geen problemen met rondwandelen
- ☐ 2 Ik heb een beetje problemen met rondwandelen
- ☐ 3 Ik heb matige problemen met rondwandelen
- ☐ 4 Ik heb ernstige problemen met rondwandelen
- ☐ 5 Ik ben niet in staat om rond te wandelen

16. Zelfzorg

- ☐ 1 Ik heb geen problemen met mijzelf te wassen of aan te kleden
- ☐ 2 Ik heb een beetje problemen met mijzelf te wassen of aan te kleden
- ☐ 3 Ik heb matige problemen met mijzelf te wassen of aan te kleden
- ☐ 4 Ik heb ernstige problemen met mijzelf te wassen of aan te kleden
- ☐ 5 Ik ben niet in staat mijzelf te wassen of aan te kleden

17. Dagelijkse activiteiten (bijv. werk, studie, huishouden, gezins- en vrijetijdsactiviteiten)

- ☐ 1 Ik heb geen problemen met mijn dagelijkse activiteiten
- ☐ 2 Ik heb een beetje problemen met mijn dagelijkse activiteiten
- ☐ 3 Ik heb matige problemen met mijn dagelijkse activiteiten
- ☐ 4 Ik heb ernstige problemen met mijn dagelijkse activiteiten
- ☐ 5 Ik ben niet in staat mijn dagelijkse activiteiten uit te voeren

18. **Pijn/klachten**

- ☐1 Ik heb geen pijn of ongemak
- ☐2 Ik heb een beetje pijn of ongemak
- ☐3 Ik heb matige pijn of ongemak
- ☐4 Ik heb ernstige pijn of ongemak
- ☐5 Ik heb extreme pijn of ongemak

19. **Angst/depressie**

- ☐1 Ik ben niet angstig of depressief
- ☐2 Ik ben een beetje angstig of depressief
- ☐3 Ik ben matig angstig of depressief
- ☐4 Ik ben erg angstig of depressief
- ☐5 Ik ben extreem angstig of depressief

Voedingsstatus

20. **Hoe groot bent u, zonder schoenen aan?**

☐☐☐ cm

21. **Bent u momenteel zwanger?**

- ☐1 Ja
- ☐2 Neen ⇨ *Ga naar Vraag 23*

22. **Hoeveel woog u zonder kleding en zonder schoenen aan vóór uw zwangerschap?**

☐☐☐ kg ⇨ *Ga naar Vraag 24*

23. **Hoeveel weegt u zonder kleding en zonder schoenen aan?**

☐☐☐ kg

Voedingsgewoonten

24. Hoe vaak eet u fruit, uitgezonderd sap geperst uit vers fruit of bereid van concentraat?

Sappen geperst uit vers fruit, bereid uit concentraat of verwerkt fruit, alsook kunstmatig gezoete sappen zijn niet inbegrepen.

- ☐ 1 1 of meer keer per dag
- ☐ 2 4 tot 6 keer per week
- ☐ 3 1 tot 3 keer per week
- ☐ 4 Minder dan 1 keer per week
- ☐ 5 Nooit

(2–5) ⇨ *Ga naar vraag 26*

25. Hoeveel porties fruit, eender welk, eet u dan elke dag?

Eén portie vers fruit komt overeen met bijvoorbeeld een appel of een banaan of twee kiwi's of zeven aardbeien of een halve pompelmoes of een halve mango.

☐☐ porties

26. Hoe vaak eet u groenten of salade, uitgezonderd aardappelen en vers sap of sap bereid van concentraat?

Zowel soepen (warm of koud) als sappen geperst uit verse groenten zijn niet inbegrepen.
Sappen bereid uit concentraat of verwerkte groenten en kunstmatig gezoete sappen ook niet.

- ☐ 1 1 of meer keer per dag
- ☐ 2 4 tot 6 keer per week
- ☐ 3 1 tot 3 keer per week
- ☐ 4 Minder dan 1 keer per week
- ☐ 5 Nooit

(2–5) ⇨ *Ga naar vraag 28*

27. Hoeveel porties groenten of salade, uitgezonderd sap en aardappelen, eet u dan elke dag?

Eén portie groenten komt overeen met bijvoorbeeld 3 opgehoopte lepels gegaarde wortelen of acht bloemkoolroosjes of een stuk komkommer van 5 cm of een middelgrote tomaat.

☐☐ porties

28. **Hoe vaak drinkt u gesuikerde frisdranken, zoals limonade of cola?**

Opgelet: 'light', 'diet', 'zero' of artificieel gesuikerde frisdranken behoren niet tot de gesuikerde frisdranken.

- ☐ 1 1 of meer keer per dag
- ☐ 2 4 tot 6 keer per week
- ☐ 3 1 tot 3 keer per week
- ☐ 4 Minder dan 1 keer per week
- ☐ 5 Nooit

(2–5) ⇨ *Ga naar vraag 30*

29. **Hoeveel gesuikerde frisdranken drinkt u gemiddeld op zo een dag?**

- ☐ 1 Minder dan 1 liter
- ☐ 2 Ongeveer 1 liter
- ☐ 3 Meer dan 1 liter

30. **Hoe vaak eet u zoete of zoute versnaperingen zoals snoep, chocolade, gebak, koekjes, roomijs, chips,...?**

- ☐ 1 1 of meer keer per dag
- ☐ 2 4 tot 6 keer per week
- ☐ 3 1 tot 3 keer per week
- ☐ 4 Minder dan 1 keer per week
- ☐ 5 Nooit

Lichaamsbeweging

De volgende vragen gaan over lichaamsbeweging. Er zullen 4 aspecten van lichaamsbeweging bevraagd worden: tijdens uw **werk**, als **transport**, in uw **vrije tijd** en uw **sedentair gedrag**.

31. Wanneer u aan het WERKEN bent, welk van het volgende omschrijft het BEST wat u doet?

Werk wordt gezien als betaald en onbetaald werk, huishoudelijke taken, voor familie zorgen, studeren of trainen.

- ☐ 1 Meestal zitten of staan
  Taken van lichte lichamelijke inspanning:
  licht kantoorwerk, lezen, schrijven, tekenen, de computer gebruiken, spreken of spreken over de telefoon, studeren, met de auto of vrachtwagen rijden, lesgeven, naaien, bakkerijproducten verkopen, haarstylen, het verkeer regelen, enz.
- ☐ 2 Meestal wandelen of taken van lichte tot matige lichamelijke inspanning
  Taken van lichte tot matige lichamelijke inspanning:
  post leveren, lichte ladingen dragen, het gazon of de tuin water geven, eeltriciteitswerken, loodgieterij, auto's repareren, machines repareren, kloppen, drillen / boren, het huis verven, verplegen, verschillende huishoudelijke taken zoals het huis poetsen en stofzuigen, winkelen, met de kinderen spelen, enz
- ☐ 3 Meetal zware arbeid of fysiek eisend werk
  Taken van zware lichamelijke inspanningen:
  zware gereedschappen gebruiken, zware bouwwerken, mijnwerken, zware lasten dragen, laden, hout hakken of stapelen, grond bewerken, scheppen of graven, spaden, tuin beplaten, enz.
- ☐ 4 Ik voer geen werktaken uit

32. In een typische week, hoeveel dagen <u>wandelt</u> u voor minstens 10 minuten aan één stuk om u <u>te verplaatsen</u>?

Bijvoorbeeld naar het werk, naar school, om te winkelen of naar de markt te gaan.

☐☐ dagen

33. Op een typische dag, hoeveel tijd spendeert u al <u>wandelend</u> om u <u>te verplaatsen</u>?

- ☐ 1 10 - 29 minuten per dag
- ☐ 2 30 - 59 minuten per dag
- ☐ 3 1 uur - 2 uur per dag
- ☐ 4 2 uur - 3 uur per dag
- ☐ 5 3 uur of meer per dag

34. In een typische week, hoeveel dagen fietst u voor minstens 10 minuten aan één stuk om u te verplaatsen?

Bijvoorbeeld naar het werk, naar school, om te winkelen of naar de markt te gaan.

☐☐ dagen

35. Op een typische dag, hoeveel tijd spendeert u al fietsend om u te verplaatsen?

- ☐ 1 10 - 29 minuten per dag
- ☐ 2 30 - 59 minuten per dag
- ☐ 3 1 uur - 2 uur per dag
- ☐ 4 2 uur - 3 uur per dag
- ☐ 5 3 uur of meer per dag

36. In een typische week, hoeveel dagen doet u sport, fitness of recreatieve (vrije tijds) activiteiten voor minstens 10 minuten aan één stuk?

Bijvoorbeeld snelwandelen, balspelen, joggen, fietsen of zwemmen.

☐☐ dagen

37. In een typische week, hoeveel tijd spendeert u in totaal aan sporten, fitnessen of recreatieve (vrije tijds) activiteiten?

☐☐ uren ☐☐ minuten

38. In een typische week, hoeveel dagen doet u aan lichaamsbeweging specifiek om uw spieren te verstevigen zoals krachttraining of verstevigingsoefeningen?

Bijvoorbeeld weerstandstraining, krachtoefeningen (met behulp van gewichtens, elastieken, eigen lichaamsgewicht ...), kniebuigingen (squats), opdrukken (push-ups), sit-ups, enz.

☐☐ dagen

39. Op een typische dag, hoeveel tijd spendeert u aan zitten of rusten?

*Bijvoorbeeld zitten aan een bureau, zitten bij vrienden, reizen in de auto, bus, trein, lezen, kaarten of tv kijken, ... maar **niet slapen**.*

☐☐ uren ☐☐ minuten

Gebruik van alcohol

40. Hoe vaak hebt u de afgelopen <u>12 maanden</u> eender welke alcoholische drank (zoals bier, wijn, cider, aperitief, cocktails, breezers, likeur, zelfgemaakte alcohol ...) gedronken?

- ☐ 1 Elke dag of bijna dagelijks
- ☐ 2 5 - 6 dagen per week
- ☐ 3 3 - 4 dagen per week
- ☐ 4 1 - 2 dagen per week

⇨ *Ga naar vraag 41*

- ☐ 5 2 - 3 dagen per maand
- ☐ 6 1 dag per maand
- ☐ 7 Minder dan 1 dag per maand

⇨ *Ga naar vraag 45*

- ☐ 8 Niet in de afgelopen 12 maanden, want ik drink geen alcohol meer
- ☐ 9 Nooit, of niet meer dan enkele slokjes in heel mijn leven

⇨ *Ga naar vraag 46*

41. Van maandag tot donderdag, op hoeveel van die 4 dagen drinkt u gewoonlijk alcoholische dranken?

- ☐ 1 Op alle 4 dagen
- ☐ 2 Op 3 dagen van de 4
- ☐ 3 Op 2 dagen van de 4
- ☐ 4 Op 1 dag van de 4
- ☐ 5 Op geen enkele van de 4 weekdagen ⇨ *Ga naar vraag 43*

42. Van maandag tot donderdag, als u alcohol drinkt, hoeveel drinkt u gemiddeld op één dag?

- ☐ 1 16 glazen of meer per dag
- ☐ 2 10 - 15 glazen per dag
- ☐ 3 6 - 9 glazen per dag
- ☐ 4 4 - 5 glazen per dag
- ☐ 5 3 glazen per dag
- ☐ 6 2 glazen per dag
- ☐ 7 1 glas per dag
- ☐ 8 Ik drink doorgaans niet tijdens de week.

43. **Van vrijdag tot zondag, op hoeveel van die 3 dagen drinkt u gewoonlijk alcoholische dranken?**

- ☐1 Op alle 3 dagen
- ☐2 Op 2 dagen van de 3
- ☐3 Op 1 dag van de 3
- ☐4 Op geen enkele van de 3 dagen van het weekend ⇨ *Ga naar vraag 45*

44. **Van vrijdag tot zondag, als u alcohol drinkt, hoeveel drinkt u gemiddeld op één dag?**

- ☐1 16 glazen of meer per dag
- ☐2 10 - 15 glazen per dag
- ☐3 6 - 9 glazen per dag
- ☐4 4 - 5 glazen per dag
- ☐5 3 glazen per dag
- ☐6 2 glazen per dag
- ☐7 1 glas per dag
- ☐8 Ik drink doorgaans niet tijdens het weekend.

45. **Hoe vaak hebt u de afgelopen 12 maanden 6 of meer glazen alcohol gedronken bij eenzelfde gelegenheid? Bijvoorbeeld wanneer u uitging, op een feestje, een maaltijd, een avond uit met vrienden of alleen bij u thuis...**

- ☐1 Elke dag of bijna dagelijks
- ☐2 5 - 6 dagen per week
- ☐3 3 - 4 dagen per week
- ☐4 1 - 2 dagen per week
- ☐5 2 - 3 dagen in een maand
- ☐6 1 dag per maand
- ☐7 Minder dan 1 dag per maand
- ☐8 Niet in de afgelopen 12 maanden
- ☐9 Nooit in heel mijn leven

Tabaksgebruik

46. Hebt u minstens 100 sigaretten (ongeveer 5 pakjes) of een gelijkwaardige hoeveelheid tabak gerookt tijdens uw leven?

- ☐ 1 Ja
- ☐ 2 Neen ⇨ *Ga naar vraag 49*

47. Rookt u <u>tegenwoordig</u>?

- ☐ 1 Ja, alle dagen
- ☐ 2 Ja, af en toe
- ☐ 3 Neen, helemaal niet

} ⇨ *Ga naar vraag 49* (bij 2 en 3)

48. Hoeveel rookt u gemiddeld elke dag?

Meerdere antwoorden mogelijk

Opgepast! Geef het aantal sigaretten, sigaren, pijpen aan, <u>niet</u> het aantal pakjes!

01.	Ik rook dagelijks	☐☐	sigaretten (zelfgerolde en/of industrieel)
02.	Ik rook dagelijks	☐☐	sigaren/cigarillos
03.	Ik rook dagelijks	☐☐	pijpen (tabak)
04.	Ik rook dagelijks	☐☐	keer chicha/waterpijp
05.	Ik rook dagelijks	☐☐	andere, omschrijf: ______________________

Elektronische sigaretten (e-sigaretten)

Een electronische sigaret, ook wel een e-sigaret of vape genoemd, of een gelijkaardig apparaat zoals een e-pijp,e-sigaar,e-shisha bootst de handeling van roken na. Deze kleine elektronische apparaten verbranden geen tabak maar verhitten een vloeistof en produceren daardoor damp. Vandaar dat elektronisch roken ook dampen genoemd wordt.

In de volgende vragen wordt met de term 'e-sigaret' naar dergelijke elektronische apparaten verwezen.

49. **Gebruikt u momenteel een e-sigaret?**

- ☐ 1 Ja, elke dag
- ☐ 2 Ja, eens of meer dan eens per week, maar niet dagelijks
- ☐ 3 Ja, eens of meer dan eens per maand, maar niet elke week
- ☐ 4 Ja, minder dan eens per maand
- ☐ 5 Nee, helemaal niet ⇨ *Ga naar vraag 51*

50. **Rookte u tabak vooraleer u voor het eerst een e-sigaret gebruikte?**

- ☐ 1 Ja
- ☐ 2 Neen

Gebruik van andere middelen

51. **Hebt u de afgelopen 30 dagen cannabis (hasjiesj of marihuana) gebruikt?**

- ☐ 1 Ja
- ☐ 2 Neen

Stress en gemoedstoestand

Hoe voelde u zich de afgelopen weken? Vermeld de situatie van dit moment, niet deze in het verleden. De laatste tijd:

52. **Hebt u zich kunnen concentreren op uw bezigheden?**

- ☐ 1 Beter dan gewoonlijk
- ☐ 2 Net zo goed als gewoonlijk
- ☐ 3 Slechter dan gewoonlijk
- ☐ 4 Veel slechter dan gewoonlijk

53. **Bent u door zorgen veel slaap tekort gekomen?**

- ☐ 1 Helemaal niet
- ☐ 2 Niet meer dan gewoonlijk
- ☐ 3 Wat meer dan gewoonlijk
- ☐ 4 Veel meer dan gewoonlijk

54. Hebt u het gevoel gehad zinvol bezig te zijn?

- ☐ 1 Zinvoller dan gewoonlijk
- ☐ 2 Net zo zinvol als gewoonlijk
- ☐ 3 Minder zinvol dan gewoonlijk
- ☐ 4 Veel minder zinvol dan gewoonlijk

55. Voelde u zich in staat om beslissingen (over dingen) te nemen?

- ☐ 1 Beter dan gewoonlijk
- ☐ 2 Net zo goed als gewoonlijk
- ☐ 3 Wat minder goed dan gewoonlijk
- ☐ 4 Veel minder goed dan gewoonlijk

56. Hebt u het gevoel gehad dat u voortdurend onder druk stond?

- ☐ 1 Helemaal niet
- ☐ 2 Niet meer dan gewoonlijk
- ☐ 3 Wat meer dan gewoonlijk
- ☐ 4 Veel meer dan gewoonlijk

57. Hebt u het gevoel gehad dat u uw moeilijkheden niet de baas kon?

- ☐ 1 Helemaal niet
- ☐ 2 Niet meer dan gewoonlijk
- ☐ 3 Wat meer dan gewoonlijk
- ☐ 4 Veel meer dan gewoonlijk

58. Hebt u plezier kunnen beleven aan uw gewone, dagelijkse bezigheden?

- ☐ 1 Meer dan gewoonlijk
- ☐ 2 Even veel als gewoonlijk
- ☐ 3 Wat minder dan gewoonlijk
- ☐ 4 Veel minder dan gewoonlijk

59. Bent u in staat geweest uw problemen onder ogen te zien?

- ☐ 1 Beter dan gewoonlijk
- ☐ 2 Net zo goed als gewoonlijk
- ☐ 3 Minder goed dan gewoonlijk
- ☐ 4 Veel minder goed dan gewoonlijk

60. Hebt u zich ongelukkig of neerslachtig gevoeld?

- ☐ 1 Helemaal niet
- ☐ 2 Niet meer dan gewoonlijk
- ☐ 3 Wat meer dan gewoonlijk
- ☐ 4 Veel meer dan gewoonlijk

61. Bent u het vertrouwen in uzelf kwijtgeraakt?

- ☐ 1 Helemaal niet
- ☐ 2 Niet meer dan gewoonlijk
- ☐ 3 Wat meer dan gewoonlijk
- ☐ 4 Veel meer dan gewoonlijk

62. Hebt u zich als een waardeloos iemand beschouwd?

- ☐ 1 Helemaal niet
- ☐ 2 Niet meer dan gewoonlijk
- ☐ 3 Wat meer dan gewoonlijk
- ☐ 4 Veel meer dan gewoonlijk

63. Hebt u zich alles bij elkaar redelijk gelukkig gevoeld?

- ☐ 1 Gelukkiger dan gewoonlijk
- ☐ 2 Even gelukkig als gewoonlijk
- ☐ 3 Minder gelukkig dan gewoonlijk
- ☐ 4 Veel minder gelukkig dan gewoonlijk

64. Voelde u zich optimistisch over uw toekomst?

- ☐1 Meer dan gewoonlijk
- ☐2 Even veel als gewoonlijk
- ☐3 Wat minder dan gewoonlijk
- ☐4 Veel minder dan gewoonlijk

Vitaliteit

65. Hoe vaak gedurende de afgelopen weken…

(Een kruisje per lijn)	Altijd	Meestal	Soms	Zelden	Nooit
01. Voelde u zich levenslustig?	☐1	☐2	☐3	☐4	☐5
02. Had u veel energie?	☐1	☐2	☐3	☐4	☐5
03. Voelde u zich uitgeput?	☐1	☐2	☐3	☐4	☐5
04. Voelde u zich moe?	☐1	☐2	☐3	☐4	☐5

Sociale contacten

66. Hoeveel personen staan u zo dichtbij dat u op hen kunt rekenen indien u met zware problemen geconfronteerd wordt?

- ☐1 Geen enkele
- ☐2 1 of 2
- ☐3 3 tot 5
- ☐4 6 of meer

67. In welke mate hebben mensen aandacht en interesse voor wat u doet?

- ☐1 Veel
- ☐2 Matig
- ☐3 Dit weet ik niet zeker
- ☐4 Weinig
- ☐5 Helemaal niet

68. Hoe gemakkelijk is het om praktische hulp van buren te krijgen als dit nodig zou zijn?

- 1 ☐ Zeer gemakkelijk
- 2 ☐ Gemakkelijk
- 3 ☐ Het is wellicht mogelijk
- 4 ☐ Moeilijk
- 5 ☐ Zeer moeilijk

Gezondheid en omgeving

69. In welke mate vormen de volgende omstandigheden een probleem in uw wijk of buurt?

(Een kruisje per lijn)	Helemaal geen probleem	Klein probleem	Redelijk groot probleem	Zeer groot probleem
01. De snelheid of de hoeveelheid van het verkeer	1 ☐	2 ☐	3 ☐	4 ☐
02. Opstapeling van vuilnis	1 ☐	2 ☐	3 ☐	4 ☐
03. Vandalisme, graffiti of opzettelijke beschadiging van eigendommen	1 ☐	2 ☐	3 ☐	4 ☐
04. Toegang tot parken of tot andere groene of recreatieve openbare terreinen	1 ☐	2 ☐	3 ☐	4 ☐

De volgende vragen hebben betrekking op omgevingskenmerken hier bij u thuis.

70. Als u denkt aan de afgelopen 12 maanden, in welke mate ergert, stoort of hindert één van de volgende condities u als u hier thuis bent?

(Een kruisje per lijn)	Helemaal niet	Een beetje	Tamelijk	Erg	Extreem
01. Luchtverontreiniging	1 ☐	2 ☐	3 ☐	4 ☐	5 ☐
02. Geurhinder afkomstig van de industrie of van andere bronnen (riolering, afval, bemesting)	1 ☐	2 ☐	3 ☐	4 ☐	5 ☐
03. Trillingen te wijten aan wegverkeer, treinverkeer, tram, vliegtuigen of ondernemingen in de buurt	1 ☐	2 ☐	3 ☐	4 ☐	5 ☐
04. Lawaai van autoverkeer	1 ☐	2 ☐	3 ☐	4 ☐	5 ☐
05. Lawaai van treinverkeer, tram of metro	1 ☐	2 ☐	3 ☐	4 ☐	5 ☐
06. Lawaai van vliegtuigen	1 ☐	2 ☐	3 ☐	4 ☐	5 ☐
07. Lawaai van ondernemingen in de buurt (fabriek, werkplaats)	1 ☐	2 ☐	3 ☐	4 ☐	5 ☐
08. Burenlawaai	1 ☐	2 ☐	3 ☐	4 ☐	5 ☐

Blootstelling aan tabaksrook

71. Hoe vaak bent u binnenshuis blootgesteld aan tabaksrook van anderen (in uw eigen woning, in de auto, op uw werkplaats, op openbare plaatsen,...)?

Enkel rook van andere personen moet in rekening worden genomen.

- ☐ 1 Alle dagen, 1 uur of meer per dag
- ☐ 2 Alle dagen, minder dan 1 uur per dag
- ☐ 3 Minstens één keer per week (maar niet elke dag)
- ☐ 4 Minder dan één keer per week
- ☐ 5 Nooit of bijna nooit ⇨ *Ga naar vraag 73*

72. Waar wordt u doorgaans binnenshuis aan tabaksrook van anderen blootgesteld?

(Meerdere antwoorden mogelijk)

- ☐ 1 Thuis
- ☐ 2 In de auto
- ☐ 3 Op de werkplaats
- ☐ 4 Op openbare plaatsen (cafés, restaurants,...)?
- ☐ 5 Andere, omschrijf: ______________________________

Informele zorgverlening

73. Staat u tenminste een keer per week niet-beroepsmatig in voor hulp of verzorging van één of meerdere personen met ouderdomsgerelateerde aandoeningen, langdurige ziekten, chronische aandoeningen of handicaps?

- ☐ 1 Ja
- ☐ 2 Neen ⇨ *Ga naar vraag 76*

74. Voor wie staat u het meest niet beroepsmatig in voor hulp of verzorging?

Slechts één antwoord mogelijk. In het geval dat er u meerdere personen helpt, selecteer diegene voor wie u het meeste verzorging biedt.

- ☐ 1 Eén of meerdere personen die tot uw huishouden behoren
- ☐ 2 Eén of meerdere familieleden die niet tot uw huishouden behoren
- ☐ 3 Eén of meerdere personen die niet tot uw huishouden of familie behoren

75. Hoeveel tijd in het totaal besteedt u gewoonlijk per week aan deze hulp of verzorging?

Sommeer de tijd die gespendeerd wordt gedurende één week aan de verzorging van alle personen (niet enkel de persoon aan wie het meeste verzorging besteed wordt)

- ☐ 1 Minder dan 10 uren per week
- ☐ 2 Tussen 10 en 20 uren per week
- ☐ 3 20 uren per week of meer

Kwetsbaarheid (enkel vanaf 50 jaar)

76. Hebt u in de laatste maand te weinig energie gehad om de dingen te doen die u wilde doen?

- ☐ 1 Ja
- ☐ 2 Neen

77. Hoe was uw eetlust in de afgelopen maand?

- ☐ 1 Vermindering van eetlust
- ☐ 2 Geen vermindering van eetlust

} ⇨ *Ga naar vraag 79*

- ☐ 3 Ik weet het niet

78. Heeft u dan meer of minder gegeten dan gebruikelijk?

- ☐ 1 Minder
- ☐ 2 Meer
- ☐ 3 Niet meer of minder

79. Omwille van een gezondheidsprobleem, hebt u moeilijkheden bij het uitvoeren van de volgende activiteiten? Houd geen rekening met moeilijkheden die volgens u niet langer dan 3 maanden zullen duren.

(Een kruisje per lijn)	Ja	Neen
01. Gewichten van meer dan 5 kilo tillen of dragen, zoals een zware boodschappentas	☐ 1	☐ 2
02. 100 meter wandelen?	☐ 1	☐ 2
03. Een trap opgaan zonder te rusten?	☐ 1	☐ 2

80. **Hoe vaak onderneemt u activiteiten die een matige inspanning vereisen, zoals tuinieren, de auto wassen, of wandelen?**

- ☐1 Meer dan één keer per week
- ☐2 Eén keer per week
- ☐3 Eén tot drie keer per maand
- ☐4 Bijna nooit of nooit

Valincidentie (enkel vanaf 65 jaar)

81. **Bent u de afgelopen 12 maanden gevallen, uitgegleden of gestruikeld waardoor u uw evenwicht verloor en op de vloer, grond of een lager gelegen niveau terechtkwam?**

- ☐1 Ja
  - → **Zo ja, hoeveel keer** ☐☐
- ☐2 Neen

Hartelijk bedankt voor uw medewerking!

Controleer even of u uw toegangscode heeft ingevuld aan het begin van deze enquête. U kan deze vragenlijst nu gratis verzenden aan de hand van de bijgevoegde omslag. Deze is reeds geadresseerd en heeft geen postzegel nodig. U hoeft hem dus enkel te versturen met de post.

Voor alle personen die te kampen hebben met sociale of psychologische problemen is er de mogelijkheid om Tele-Onthaal te contacteren. Bij deze organisatie kunt u in alle anonimiteit uw problemen vertellen en wordt er een luisterend oor aangeboden. De dienst is 24 uur op 24 en 7 dagen op 7 bereikbaar via het gratis nummer 106. U kunt ook chatten met een vrijwilliger via hun website. Meer informatie kunt u vinden op hun website: http://www.tele-onthaal.be/.
